# Large-scale extraction of accurate drug-disease treatment pairs from biomedical literature for drug repurposing

**DOI:** 10.1186/1471-2105-14-181

**Published:** 2013-06-06

**Authors:** Rong Xu, QuanQiu Wang

**Affiliations:** 1 Medical Informatics Division, Case Western Reserve, Cleveland, OH, USA; 2 ThinTek LLC, Palo Alto CA, USA

## Abstract

**Background:**

A large-scale, highly accurate, machine-understandable drug-disease treatment relationship knowledge base is important for computational approaches to drug repurposing. The large body of published biomedical research articles and clinical case reports available on MEDLINE is a rich source of FDA-approved drug-disease indication as well as drug-repurposing knowledge that is crucial for applying FDA-approved drugs for new diseases. However, much of this information is buried in free text and not captured in any existing databases. The goal of this study is to extract a large number of accurate drug-disease treatment pairs from published literature.

**Results:**

In this study, we developed a simple but highly accurate pattern-learning approach to extract treatment-specific drug-disease pairs from 20 million biomedical abstracts available on MEDLINE. We extracted a total of 34,305 unique drug-disease treatment pairs, the majority of which are not included in existing structured databases. Our algorithm achieved a precision of 0.904 and a recall of 0.131 in extracting all pairs, and a precision of 0.904 and a recall of 0.842 in extracting frequent pairs. In addition, we have shown that the extracted pairs strongly correlate with both drug target genes and therapeutic classes, therefore may have high potential in drug discovery.

**Conclusions:**

We demonstrated that our simple pattern-learning relationship extraction algorithm is able to accurately extract many drug-disease pairs from the free text of biomedical literature that are not captured in structured databases. The large-scale, accurate, machine-understandable drug-disease treatment knowledge base that is resultant of our study, in combination with pairs from structured databases, will have high potential in computational drug repurposing tasks.

## Background

### Computational drug repurposing approaches

Drug repurposing, the use of known drugs to treat new diseases, has been growing in importance in the last few years [1,2] because of the prohibitively high cost of drug development, as well as its increasing failure rate. Many computational strategies for drug repurposing have been published [3]. These approaches include repositioning based on chemical similarity [4,5], molecular activity similarity [6,7], molecular docking [8], gene expression similarity [9,10], and drug side effect similarity [11]. Recently, Chiang *et al* proposed a data-driven approach to using FDA-approved drug-disease treatment associations for drug repurposing [12]. Even though Chiang’s study used only FDA-approved drug-disease pairs, the researchers were able to infer novel drug uses based on shared treatment profile using a network-based, guilt-by-association method.

A vast amount of drug-disease treatment information exists in the large corpus of published biomedical literature, especially in published clinical trial studies and case reports. Currently, there are 591,623 clinical trial reports and 1,554,544 clinical case reports available on MEDLINE. The drug-disease relationships in biomedical literature include FDA-approved, experimental, and unsuccessful or failed associations. In the USA, and many other countries, off-label use of prescribed drugs are common [13] and many of these off-label new drug usage results have published in clinical case reports. Consider the following sentence from a clinical case report: “**Imatinib** in the treatment of **follicular dendritic sarcoma**: a case report and review of literature." (PMID 17596748). This sentence contains drug repurposing information of using imatinib to treat follicular dendritic sarcoma, for which surgery and radiotherapy are considered as the mainstay treatment options. Another clinical case study example is the repurposing of gabapentin, an FDA-approved drug for controlling seizures in patients with epilepsy, to treat patients with tinnitus, as shown in sentence: “**Gabapentin** for the treatment of **tinnitus**: a case report” (PMID 11233342). In this study, we develop a large-scale, pattern-based relationship extraction algorithm to extract drug-disease treatment pairs from published biomedical literature. These pairs include FDA-approved, experimental, and even failed drug-disease associations (the reasons behind failed drug indications are important for drug repurposing). Currently, there exists no knowledge base for failed drug-disease associations.

A large-scale and accurate list of drug-disease treatment pairs derived from published biomedical literature can be used for drug repurposing in two ways: first, the extracted pairs themselves contain many interesting drug-disease repurposing pairs with evidence from case studies or small-scale clinical studies (as shown above). Second, these pairs can be used in network-based systems approaches for drug repurposing. For example, if drug 1 is similar to drug 2 (similarity can be measured based on shared genes, pathways, gene expression profiles, chemical structures or phenotypes), and disease 1 can be treated by drug 1 (based on drug-disease relationship), then we can hypothesize that disease 1 can also be treated by drug 2. This is a very simple example and we can add more constraints to the repurposing algorithms, but drug-disease relationships will be important to connect drugs to diseases.

### Drug-disease relationship extraction from biomedical literature

Currently, more than 20 million biomedical abstracts are available on MEDLINE, making it a rich source of biomedical information, including drug-disease treatment associations. However, despite the sheer volume of published articles, most of the available knowledge is buried in free text with limited machine understandability. Common approaches for relation extraction use rule-based, statistical approaches, machine learning or natural language processing (NLP) techniques [14-18]. Automatically extracting drug-disease treatment relationships from free text is an active research area. Cimino et al. used MeSH descriptors and co-occurrence statistics to generate semantic relation extraction rules in order to detect relations in MEDLINE article titles [19]. Lee et al. and Abacha et al. applied manually built patterns to identify treatment specific relations between drugs and diseases [20,21]. Rosario et al. classified seven relation types, including drug-disease treatment type, using generative and neural network models [22]. Chen et al. used co-occurrence statistics to rank the association between eight disease and relevant drugs[23]. Rindflesch et al. developed the SemRep system to identify semantic relations in the biomedical literature based on linguistic analysis of text and domain knowledge [24]. Recently, Neveol et al. automatically extracted and integrated drug indication information from multiple resources [25]. To extract drug-disease relationships from biomedical text, the researchers use MeSH terms to retrieve related articles from which drug-disease treatment pairs are then extracted. Many of the above studies leveraged MeSH terms in order to extract treatment-specific drug-disease pairs. However, not all drug-disease treatment pairs were captured by MeSH terms. For the two drug repurposing case studies mentioned previously: “imatinib-follicular dendritic sarcoma” and “gabapentin-tinitus”, neither of the pairs are specified in MeSH headings. Machine-learning approaches have been applied to extract drug-disease treatment pairs from free text. Bundschus et al. developed a conditional random fields method to identify the semantic relations between diseases and treatments [26]. The researchers trained and tested the model on a manually annotated text corpus consisting of 3570 sentences generated from MEDLINE 2001 abstracts and reported a 79.5% accuracy in identifying treatment semantic relationship. Similarly Islamaj Dogan et al. developed a context-blocks model for identifying clinical relationships, including treatment semantic relationship, in patient records. The model was trained and tested on a set of 826 patient records and achieved a F-score of 0.704 in identifying drug-disease treatment relationship. Even though both studies reported high performance in identifying treatment semantic relationship from manually annotated test dataset, it is still unknown if these models are generalizable and if they can achieve the same high performance when tested on all MEDLINE abstracts using all known drug-disease treatment pairs (eg., pairs extracted from FDA drug labels or pairs from ClinicalTrials.gov) as test data.

In this study, we develop a large-scale pattern-based approach to extracting drug-disease treatment associations from 20 million MEDLINE articles. Unlike previous studies, our study does not rely on MeSH terms or manually annotated training datasets to classify extracted drug-disease pairs and requires minimal human effort. While most relationship extraction methods put equal emphases on precision and recall, our study focuses on building a large scale and accurate drug-disease treatment relationship knowledge base for the purpose of ‘in silico; drug target discovery and drug-repurposing; therefore high precision, large-scale (not necessary high recall) and unbiasedness are important. The assumption underlying our pattern-learning approach is that even though treatment-specific semantic relationship between a drug and a disease can be expressed in many different ways due to the flexibility and expressive nature of human natural language, these patterns are not randomly distributed. There exist predominant patterns that people are commonly used to describe treatment-specific drug-disease associations, such as “DRUG in the treatment of DISEASE” and “DRUG for the treatment of DISEASE.” In fact, searching MEDLINE for the phrase “in the treatment of,” we retrieved more than 250,000 sentences. Searching for a more specific phrase “in the treatment of breast cancer,” we retrieved more than 1500 sentences. The drugs used to treat breast cancer include tamoxifen, dibromodulcitol, trastuzumab, lapatinib, vindesine, letrozole among many others. Of these drugs, only a few are FDA-approved. In this study, we first automatically learn treatment-specific textual patterns using known drug-disease pairs. We then extract additional drug-disease pairs from published biomedical literature using these learned patterns.

## Data and methods

**Figure 1:**
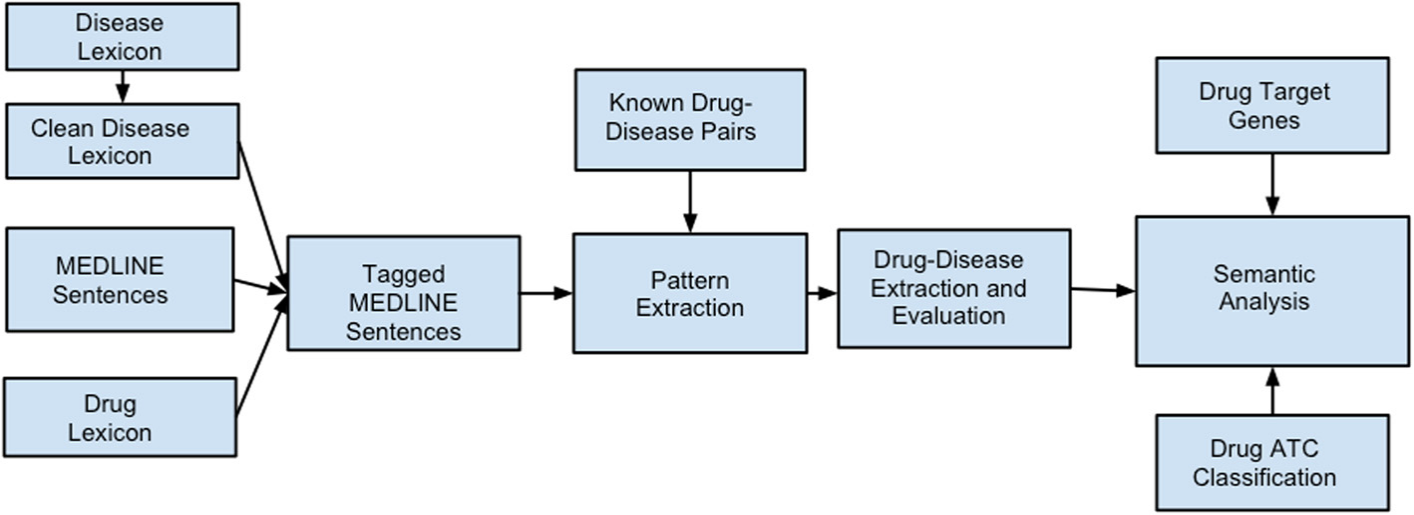
**Flow chart of the overall pattern learning, pair extraction and semantic analysis process.** The entire process consisted of the following steps: (1) obtain entire MEDLINE corpus, (2) create a clean and MEDLINE-specific disease lexicon, (3) tag MEDLINE sentences with drug and disease entities, (4) extract treatment-specific patterns from tagged MEDLINE sentences using known drug-disease pairs, (5) extract new drug-disease pairs from tagged MEDLINE using selected patterns, (6) semantic analysis of extracted drug-disease pairs.

The entire experimental process consistes the following steps: (1) obtain and parse entire MEDLINE corpus; (2) create disease and drug lexicons; (3) tag MEDLINE sentences with drug and disease entities; (4) Find treatment specific patterns; (5) extract additional pairs from MEDLINE with selected patterns; and (6) perform semantic analysis of extracted drug-disease pairs (Figure 1).

### Obtain MEDLINE data

We have used 20 million MEDLINE abstracts (roughly 100 million sentences) published from 1965 to 2010 as the text corpus for our task of treatment-specific drug-disease relationship extraction. The 2010 MEDLINE/PubMed baseline XML files was downloaded from NLM’s anonymous FTP server at ftp://ftp.nlm.nih.gov/nlmdata/.medleasebaseline/. The MEDLINE XML files were then parsed. Abstracts and titles were extracted and split into sentences.

### Create drug and disease lexicons

**Figure 2:**
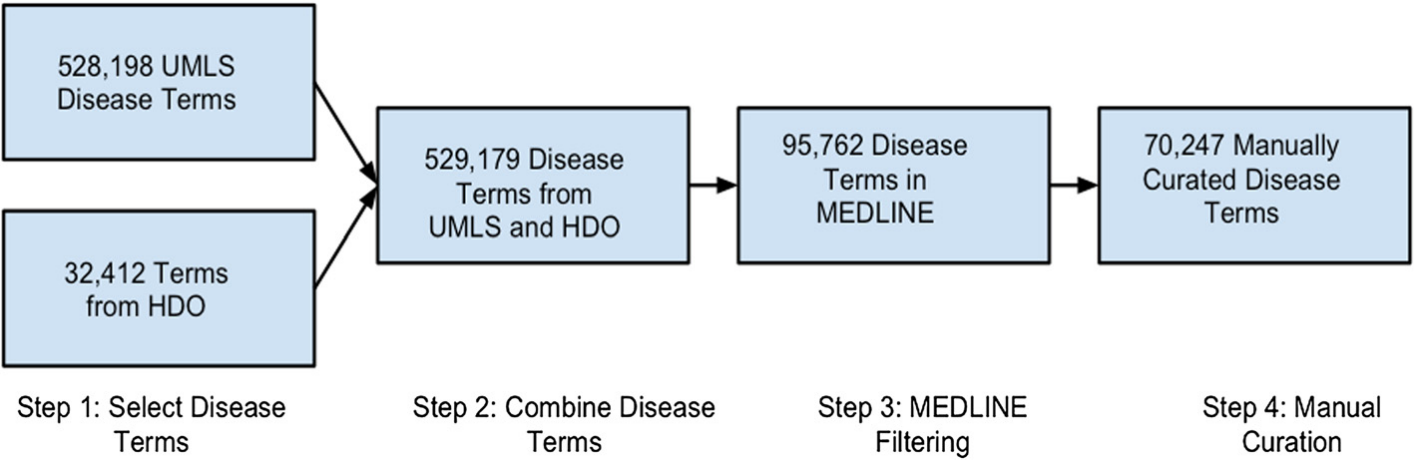
The process in creating a clean and MEDLINE specific disease lexicon.

**Clean and MEDLINE-specific disease lexicon**: Highly accurate and comprehensive lexicons are prerequisites for many biomedical relationship extraction tasks, including our task of extracting drug-disease pairs from MEDLINE. In this study, we created a clean and MEDLINE-specific disease lexicon by combining an automatic approach and manual curation (Figure 2). The disease lexicon is based on the UMLS (Unified Medical Language System) Metathesaurus (2009 AB version) and Human Disease Ontology (HDO). We first created a disease lexicon of 528,198 distinct terms by combining UMLS terms with following semantic types: “Disease Or Syndrome,” “Neoplastic Process,” “Sign Or Symptom,” “Congenital Abnormality,” “Mental or Behavioral Dysfunction,” and “Anatomical Abnormality.” We then added 32,414 distinct terms from HDO (http://bioportal.bioontology.org/ontologies/1009). The initial disease lexicon consisted of 529,179 distinct terms. Since our task in this study is to extract drug-disease relationship from MEDLINE, we are only interested in disease terms that have appeared in MEDLINE at least once. One of our previous studies has shown that many UMLS terms have never appeared in MEDLINE [27]. In order to build a MEDLINE-specific disease lexicon as well as to reduce our manual curation effort, we tagged all 20 million MEDLINE abstracts with terms from the initial disease lexicon. We then filtered out terms with MEDLINE frequency of zero. After this MEDLINE filtering, the disease lexicon consisted of 95,762 terms, a 82% reduction from original lexicon. We then manually curated the disease lexicon by removing non-disease terms (ie, brain, liver etc), ambiguous disease terms (ie consumption, weak etc) and terms that were too general (ie disorder, disease, deficiency etc). The final curated disease lexicon consisted of 70,247 terms.

**Drug lexicon**: The drug lexicon was downloaded from (http://www.drugbank.ca/) and consisted of 6,516 drugs, including both FDA-approved drugs and experimental drugs. The decision of using drug names from DrugBank instead of RxNORM or other sources is that DrugBank contains both experimental and FDA-approved clinical drugs.

### Extract known drug-disease pairs from Clinicaltrials.gov

ClinicalTrials.gov is a registry of federally and privately supported clinical trials conducted in the United States and around the world. For each of the trials listed at ClinicalTrials.gov, there is associated medical condition and drug treatment information. We downloaded a total of 115,026 clinical trial XML files from Clinicaltrials.gov (data accessed in 04/2011). A total 196,002 drug-disease pairs were extracted from the downloaded XML files. Many of the disease and drug names in the drug-disease pairs were in free text form. In addition, drug names are often mixtures of drug brand names and trade names. We performed named entity recognition for both drug and disease terms. We then mapped drug trade names to their generic names. Drug generic names as well as trade names were downloaded from DrugBank. After these steps, total 52,000 drug-disease pairs were obtained. These pairs were subsequently used as input (or seeds) to learn treatment-specific patterns, which then were used to extract additional drug-disease pairs from MEDLINE.

### Tag MEDLINE sentences and extract patterns

We tagged MEDLINE sentences with disease entities from the clean disease lexicon and drug entities from the drug list we extracted from DrugBank. The tagging was based on case-insensitive extact string matching for high precision an d efficiency. For each sentence tagged with both drug and disease entities, we extracted the textual patterns between each pair. The pattern could be “DRUG pattern DISEASE” if the drug entity precedes the disease entity or “DISEASE pattern DRUG” vice versa. For example, from the phrase: “Role of **irinotecan***in the treatment of***small cell carcinoma**” (PMID: 11995707), we extracted the pattern “DRUG *in the treatment of* DISEASE.” From the sentence: “Seventeen women with **breast cancer***were treated with***tamoxifen** (20 mg, twice a day)” (PMID 06798066), the pattern “DISEASE *were treated with* DRUG” was extracted.

### Find treatment-specific patterns

Drug-disease pairs from ClinicalTrials.gov were first used as input to learn drug-disease treatment-specific patterns. Then the learned patterns were used to extract additional pairs from MEDLINE. For example, using the pairs from ClinicalTrial.gov, we learned a treatment-specific pattern “DRUG in the treatment of DISEASE”. We then used this learned pattern to extract additional drug-disease pairs from MEDLINE, which were not included in ClinicalTrials.gov. If the pattern “DRUG in the treatment of DISEASE” is associated with 1,000 pairs from ClinicalTrials.gov and 10,000 pairs in MEDLINE, then we will extract an additional 9,000 pairs from MEDLINE using this pattern.

The patterns between drug entities and disease entities are often highly complicated. The patterns can be very general such as “DRUG and DISEASE” or very specific such as “DRUG *in combination with 5-FU/leucovorin (LV) was subsequently evaluated as first-line therapy for* DISEASE” as shown in the sentence “**Irinotecan***in combination with 5-FU/leucovorin (LV) was subsequently evaluated as first-line therapy for***metastatic colorectal cancer** in two randomized, phase III studies” (PMID 11585970). In addition, the patterns between a drug entity and a DISEASE entity are often unrelated to drug treatment. For instance, the pattern “DRUG*-induced* DISEASE” in sentence “**Tamoxifen***-induced***endometrial cancer**” (PMID 12701962) is related to drug side effect. In order to find drug treatment specific patterns, we extracted the textual patterns between known drug-disease pairs from Clinicaltrials.gov. We then ranked the patterns by the number of associated known drug-disease pairs. Finally, we manually examined the top patterns and selected drug treatment specific ones. After the ranking step, the time required to examine the top ranked patterns was minimal (less than 10 minutes).

### Extract additional pairs from MEDLINE with selected patterns

For each of the manually selected treatment-specific patterns, we extracted its associated drug-disease pairs from tagged MEDLINE sentences. These patterns were learned using known drug-disease pairs. Here, we used them to extract additional drug-disease pairs from MEDLINE.

### Evaluate extracted drug-disease pairs

In order to evaluate drug-disease pairs extracted from MEDLINE, which include FDA-approved as well as experimental drug-disease pairs, we manually created two MEDLINE-specific datasets to evaluate the precision and recall of the extraction algorithm. The first evaluation set consisted of drug-disease treatment pairs for the drug “irinotecan”. The second set consisted of drug-disease pairs for the disease “thrombocytopenia”. To create the “Irinotecan-Disease” evaluation set, we first retrieved all MEDLINE sentences (not just sentences containing the patterns) tagged with the term “irinotecan” and at least one disease term. We then manually extracted 360 treatment-specific pairs from these sentences. For creating the evaluation set “Drug-Thrombocytopenia”, we retrieved all MEDLINE sentences tagged with thrombocytopenia and at least one drug term. We manually extracted 43 treatment specific pairs from those sentences. The annotation task was performed by three curators. Each curator independently annotated tagged sentences and created two evaluation sets. Only the pairs agreed upon by all three curators were used in the final evaluation. The two sets were created independent of the methods we used (evaluators did not know the patterns we used). In this way, the final performance captured the effect of both the learned patterns and the quality of the drug and disease lexicons. Standard precision, recall, and F1 measures were used to evaluate extracted drug-disease pairs. One of the limitations is that these two manually created evaluation datasets (one drug and one disease only) may not be representative for other diseases and drugs. However, due to the intensive manual curation, we did not create evaluation datasets for multiple drugs and multiple diseases. Since the aim of this paper is to extract many additional pairs (pairs that are not included in ClinicalTrials.gov) from MEDLINE, we could not use pairs from ClincialTrials.gov to evaluate these additional pairs extracted from MEDLINE. But we did used pairs from ClinicalTrials.gov as prior knowledge (or seeds) to learn treatment-specific patterns.

### Semantic analysis of extracted drug-disease pairs

To demonstrate the potential of the drug-disease pairs that we extracted from MEDLINE using the selected patterns in drug repurposing, we studied the correlations of our extracted drug-disease pairs with drug target genes as well as drug therapeutic classes. We extracted 10,478 drug-target gene pairs from DrugBank (accessed in 01/2012) and extracted 5,544 drug-ATC associations from the World Health Organization Anatomical Therapeutic Chemical (ATC) Classification System (http://www.whocc.no/atc). Examples of these associations include *tamoxifen-anti-estrogens* and *trometamol-hemofiltrates*. For all drug-drug pairs that shared disease indications, we calculated the average shared target genes as well as shared ATC codes, then compared them to those of all drug-drug pairs.

## Results

### Analyze patterns associated with known drug-disease pairs

**Figure 3:**
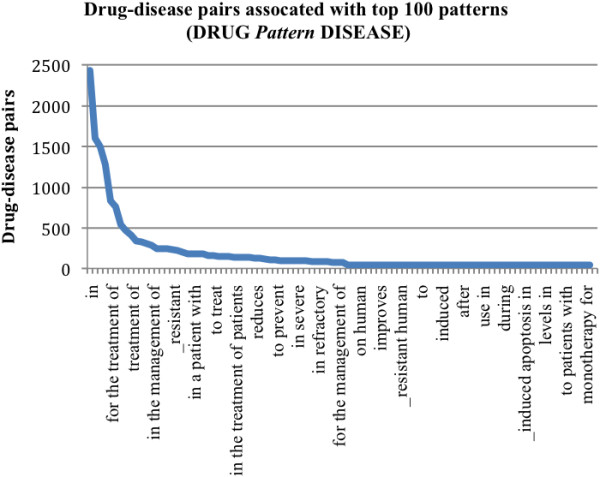
**Top 100 “*DRUG Pattern DISEASE*” patterns and associated pairs.** The distribution of top 100 patterns along with the numbers of their associated drug-disease pairs. The textual patterns are in the format of “DRUG pattern DISEASE” where the drug entity precedes the pattern and the SE entity follows the pattern. Examples include “irinotecan in the treatment of colorectal cancer”.

**Figure 4:**
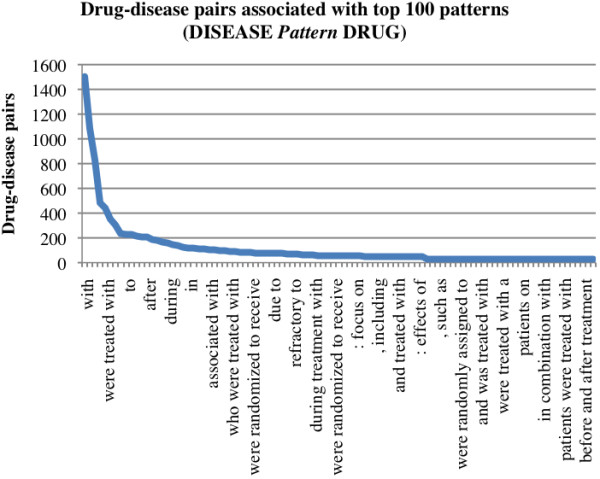
**Top 100 “ *DISEASE Pattern DRUG *” and associated pairs.** The distribution of top 100 patterns along with the numbers of their associated drug-disease pairs. The textual patterns are in the format of “DISEASE pattern DRUG” where the disease entity precedes the pattern and the drug entity follows the pattern. Examples include “breast cancer was treated with tamoxifen”.

Among 52,066 drug-disease pairs extracted from Clinicaltrials.gov XML files, 11,489 pairs co-occurred in MEDLINE sentences. From these pairs, we extracted 339,746 unique textual patterns in the format “DRUG Pattern DISEASE” and 173,738 patterns in the form “DISEASE Pattern DRUG”. Among these patterns, 501,331 (97.6%) were associated with only one drug-disease pair in the entire MEDLINE data collection. The distributions of the top 100 patterns are shown in Figure 3 and Figure 4. As seen in these two figures, drug-disease pairs are more often specified in the form of “DRUG pattern DISEASE” than in “DISEASE pattern DRUG”. In addition, top patterns in the form “DRUG pattern DISEASE” (e.g., “DRUG *in the treatment of* DISEASE”) are more specific than patterns in the form of “DISEASE pattern DRUG” (e.g., “DISEASE *with* DRUG”). This is largely due to the fact that our algorithm only extracted text patterns between drug and disease entities, and ignored patterns surrounding the pairs. For example, the pattern “*treat* DISEASE *with* DRUG”, instead of “DISEASE *with* DRUG” is a treatment-specific pattern. Extracting patterns surround the drug-disease pairs will involve pattern structure determination and boundary detection. In the future, we will incorporate phrase structures into the pattern extraction process. However, we do believe that simple textual patterns coupled with the large amount of data (data redundancy) will get us pretty far in extracting many drug-disease pairs from MEDLINE. Among the top 100 ranked patterns, many are treatment-specific, such as “DRUG *in the treatment of* DISEASE,” “DRUG *treatment of* DISEASE,” “DRUG *for the therapy for* DISEASE” and “DISEASE *were treated with* DRUG.” In addition, these patterns are not randomly distributed as shown in Figure 3 and 4.

### Extract additional pairs from MEDLINE using selected patterns

**Figure 5:**
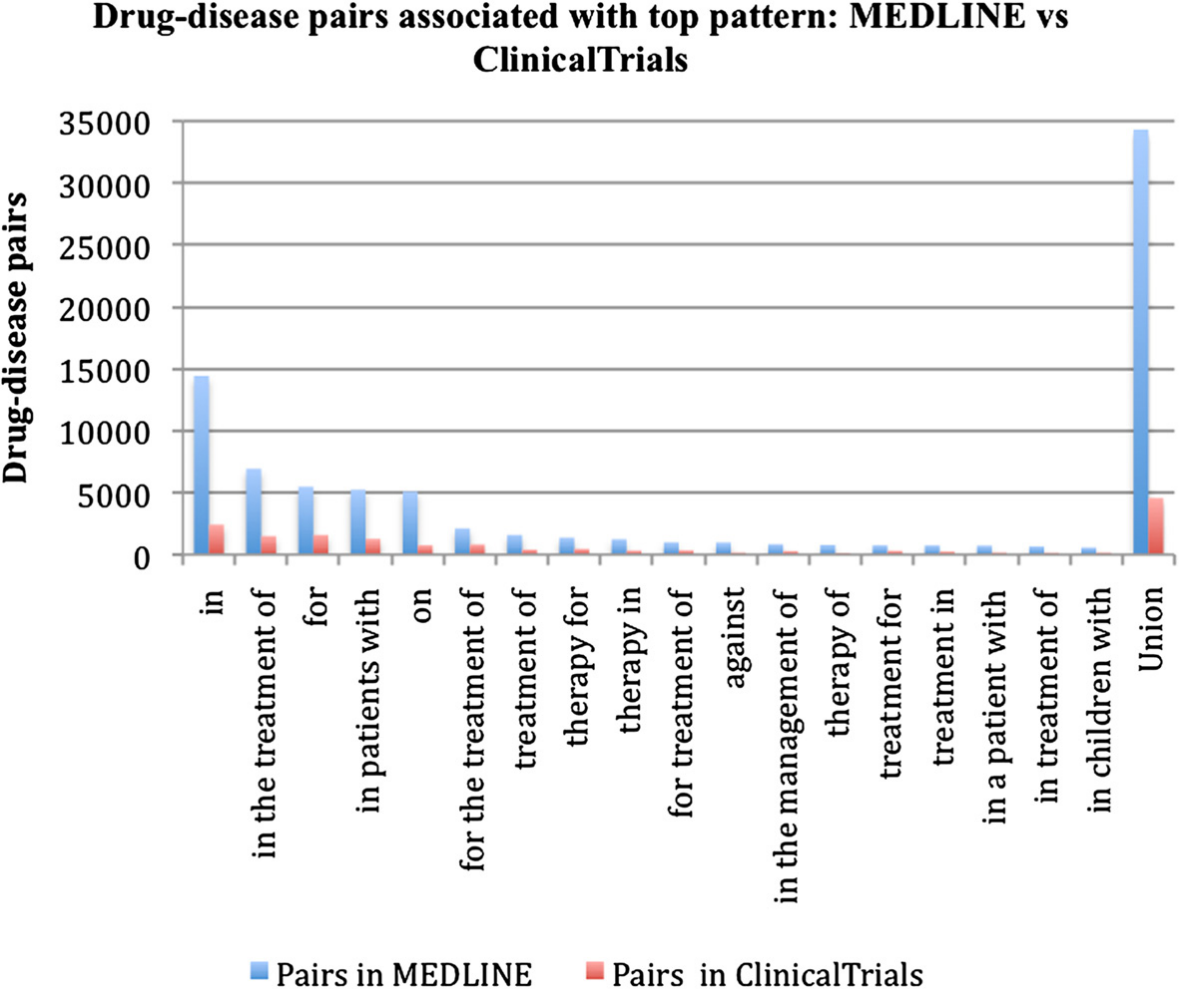
**Drug-disease pairs associated with selected patterns (MEDLINE vs. Clinicaltrials.gov).** Number of additional drug-disease pairs extracted from MEDLINE using each selected pattern. For each pattern, the blue bars show the numbers of associated drug-disease pairs in ClinicalTrials.gov. The red bars show the number of associated drug-disease pairs in MEDLINE. The difference shows the potentially additional pairs associated with each pattern.

We manually examined the top 100 ranked patterns in the format of “DRUG pattern DISEASE” and selected 17 treatment-specific patterns. This manual examination took less than 10 minutes. These patterns are: DRUG (*in, in the treatment of, for, in patients with, for the treatment of, treatment of, therapy for, therapy in, for treatment of, against, in the management of, therapy of, treatment for, treatment in, in a patient with, in treatment of, in children with*) DISEASE. We ignored the patters in the form of “DISEASE pattern DRUG” since they are less specific and associated with fewer drug-disease pairs. Using the selected patterns, we extracted many additional drug-disease pairs from MEDLINE sentences (Figure 5). For instance, using the pattern “DRUG *in* DISEASE”, we extracted a total of 14,400 distinct drug-disease pairs from MEDLINE. Among them, only 2,431 were pairs from ClinicalTrials.gov. Similar trends were observed for all other patterns. In summary, from the selected 17 patterns, we extracted 34,306 unique drug-disease pairs from MEDLINE. This is a more than six fold increase compared to their associated 4,535 known pairs extracted from ClinicalTrials.gov. Drug-disease pairs extracted from MEDLINE combined with known pairs from ClinicalTrials.gov provide a more comprehensive treatment-specific knowledge base for drug repurposing. In this study, we only selected 17 patterns in the form of “DRUG pattern DISEASE”. In order to build a more comprehensive drug-disease relationship knowledge base, we may need select more patterns, including patterns in the form of “DISEASE pattern DRUG”.

### Precision and recall evaluation of the extracted drug-disease pairs

We used two manually curated evaluation datasets to measure precisions and recalls of extracted drug-disease pairs. The first evaluation dataset consisted of 360 drug-disease treatment pairs for the drug irinotecan. Among these 360 irinotecan-disease goldstandard pairs, 132 pairs (36.7%) appear in MEDLINE sentences only once. Examples of these uncommon pairs include *irinotecan-uterine cervical cancer*, *irinotecan-relapsed rhabdomyosarcoma* and *irinotecan-thymic mucoepidermoid carcinoma*. The second evaluation dataset consisted of 43 pairs for disease thrombocytopenia, among which 9 pairs (20.9%) appeared in MEDLINE sentences only once. Using the 17 selected treatment-specific patterns, we extracted a total of 52 irinotecan-disease pairs from MEDLINE sentences. When evaluated using the irinotecan-disease evaluation dataset, we achieved a precision of 0.904 and a recall of 0.131. Similarly, we extracted 10 drug-thrombocytopenia pairs using the selected patterns, with a precision of 0.800 and recall of 0.186 when evaluated using the drug-thrombocytopenia evaluation dataset (Table 1). As is the case for many pattern-based relationship extraction approaches, our algorithm achieved high precisions but relatively low recalls. If a drug-disease pair appeared in MEDLINE only once, the chance of it being associated with any of the selected patterns was small. We then investigated whether the algorithm had higher recalls for common pairs. We calculated the recalls of the algorithm with different MEDLINE frequency cutoffs (the overall precisions did not change at different cufoffs). As shown in Table 1, the algorithm had much better recalls in extracting more frequent pairs. For irinotecan-disease pairs appearing in MEDLINE five or more times, the algorithm achieved a recall of 0.509. The recall increased to 0.842 in extracting pairs appearing in MEDLINE 30 or more times. Similarly, the recall increased from 0.186 for all drug-thrombocytopenia pairs to 0.667 for pairs appearing in MEDLINE 30 or more times. In summary, the pattern-based relationship extraction algorithm yields high precisions. The recalls of the algorithm depend upon the pair frequency and increase as the MEDLINE frequency increases. In summary, this pattern-based relationship extraction approach using a few selected patterns is able to accurately extract most common drug-disease pairs from MEDLINE.

**Table 1:** Precision, recall and F1 values at different frequency cutoffs

**GoldStandard**	**MEDLINE**	**Precision**	**Recall**	**F1**
	**Frequency**			
Irinotecan-Disease	>=1	0.904	**0.131**	**0.228**
	>=5	0.904	0.357	0.512
	>=10	0.904	0.509	0.651
	>=20	0.904	0.710	0.795
	>=30	0.904	**0.842**	**0.872**
Drug-Thrombocytopenia	>=1	0.800	**0.186**	**0.302**
	>=5	0.800	0.333	0.471
	>=10	0.800	0.429	0.558
	>=20	0.800	0.500	0.615
	>=30	0.800	**0.667**	**0.727**

The precisions, recalls and F1 values of extracted drug-disease pairs at different frequency cutoffs. Two evaluation datasets were used: Irinotecan-Disease and Drug-Thrombocytopenia. The precision, recall and F1 for all extracted pairs (frequency >=1) evaluated using Irinotecan-Disease dataset is 0.904, 0.131 and 0.228, respectively.

### Semantic analysis of extracted drug-disease pairs

**Figure 6:**
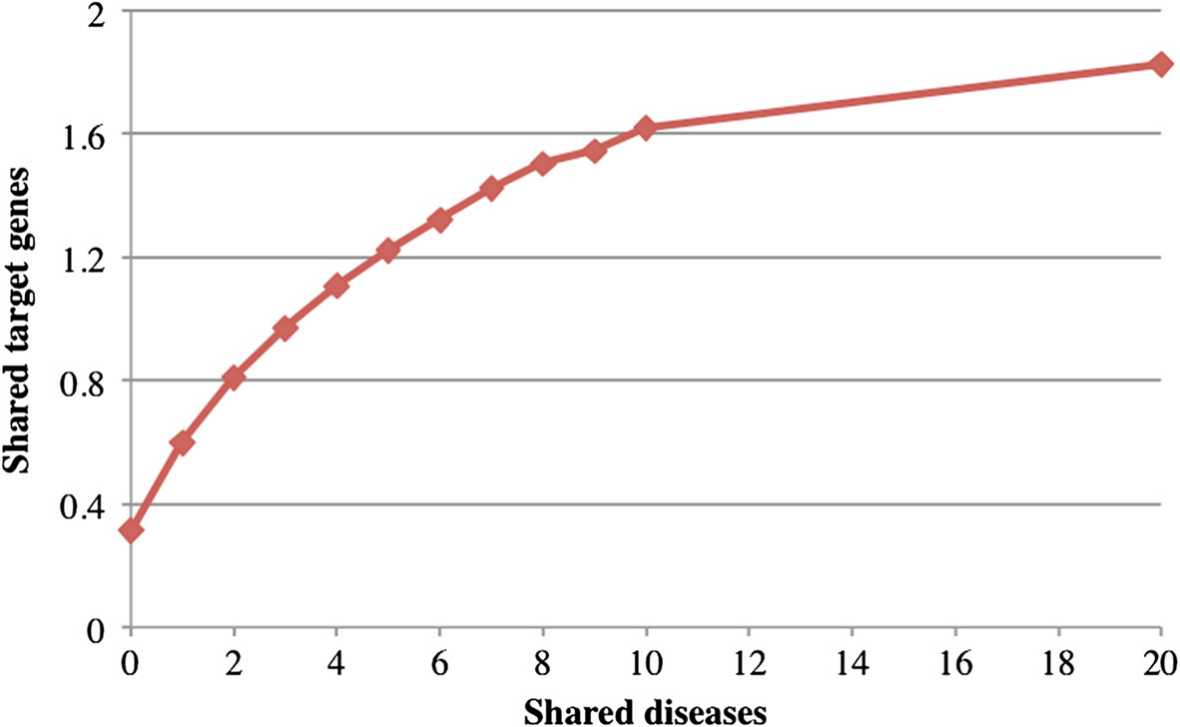
**Average number of shared target genes for drug-drug pairs sharing diseases.** The average shared target genes is 0.312 for all drug-drug pairs (shared disease >=0) and 1.691 for pairs sharing >=10 diseases. The number of shared target genes increases as the number of shared diseases increases.

**Figure 7:**
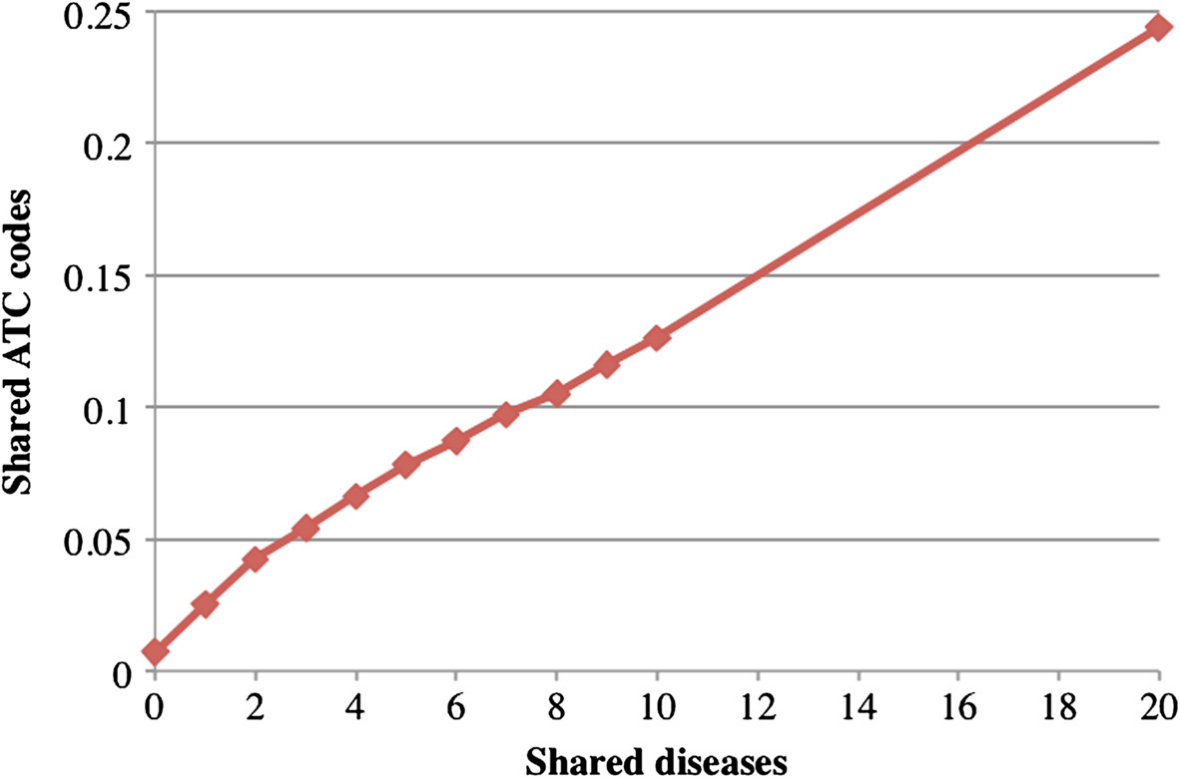
**Average number of shared ATC codes for drug-drug pairs sharing diseases.** The average shared ATC codes is 0.004 for all drug-drug pairs (shared disease >=0) and 0.126 for pairs sharing >=10 diseases. The number of shared ATC codes increases as the number of shared diseases increases.

Next, we investigated the correlations between extracted drug-disease pairs and drug target genes as well as with drug therapeutic classes. We limited the drugs to those appearing in both extracted drug-disease pairs and drug target gene association pairs or drug-ATC code associations. For every drug-drug pair, we computed the number of shared diseases and shared target genes or ATC codes. The average number of shared target genes is 0.312 for all drug-drug pairs. The number increased to 0.597 for drug-drug pairs sharing at least one disease and to 1.691 for pairs sharing 10 or more diseases (Figure 6). The average number of shared ATC is 0.004 for all drug-drug pairs and 0.007 for pairs sharing at least one disease (Figure 7). The number increased to 0.126 for drug-drug pairs sharing 10 or more diseases. In summary, the extracted drug-disease pairs have strong associations with both drug targets and drug treatment classes, and therefore have high potential for drug repurposing.

## Discussion

In this study, we developed a pattern-based relationship extraction method to mine drug-disease treatment associations from 20 million published MEDLINE abstracts. We extract total of 34,305 unique drug-disease pairs, the majority of which are not captured in any existing structured databases. The precision and recall are 0.904 and 0.131 respectively for all pairs, and 0.904 and 0.842 respectively for frequent pairs.

Even though our algorithm has achieved high precisions and extracted a large number of additional drug-disease treatment pairs from MEDLINE abstracts, there are several limitations to our study: (1) We only used the simple patterns “DRUG pattern DISEASE”. The recall of such a pattern critically depends on the coverage of the underlying lexicon. In our future studies, we will experiment two additional patterns: (a) “NP1 pattern NP2” where NP1 and NP2 are noun phrases; and (b) “NP1 pattern NP2” where NP1 and NP2 are noun phrases. NP1 contains a drug term and NP2 contains a disease term. Our current approach does not use syntactic information, and its precision and recall depend on the underlying lexicons. Both patterns (a) and (b) rely on parser information to reduce the number of patterns extracted and to increase recall by extracting pairs whose substrings are contained in the input lexicons. For example, in the sentence, “The effect of irinotecan in the treatment of metastatic and recurrent colorectal cancer,” the term “colorectal cancer” instead of “metastatic and recurrent colorectal cancer” is included in the disease lexicon. Using the pattern “in the treatment of”, both pattern (a) and pattern (b) will extract the correct drug-disease pair “irinotecan-metastatic and recurrent colorectal cancer”, but our current method will not, since the term “colorectal cancer” instead of “metastatic and recurrent colorectal cancer” is included in the lexicon. (2) This pattern-based method is limited to extracting pairs from sentences only, not from abstracts. Though important pairs often appear in sentences, some drug-disease pairs may appear only in abstracts. In order to extract drug-disease pairs from abstracts, other relationship extraction methods will be necessary. However, as the size of text corpus increases, the likelihood that drug-disease pairs will appear in a sentence will increase. (3) Even though we extracted 34,305 unique drug-disease pairs using only 17 selected top patterns, the top patterns may only capture common drug-disease pairs. If a drug-disease pair appears in MEDLINE only once, the likelihood of it being associated with one of the selected top patterns is small. In order to increase the recall, we can increase the number of selected patterns, develop other algorithms to complement the pattern-based approach, or increase the size of the text corpus to include full-text articles, web pages or electronic patient medical records. (4) Highly accurate and comprehensive lexicons are prerequisites for many biomedical relationship extraction tasks, including our task of extracting drug-disease pairs from MEDLINE. For drug-disease treatment relationship extraction from MEDLINE, we can obtain a list of accurate FDA-approved drugs with reasonable coverage from DrugBank, or PharmGKB. However, obtaining a disease list with both good accuracy and coverage for this specific task is more challenging. The precisions and recalls of using UMLS-based lexicons in extracting diseases from biomedical text vary [28,29]. In this study, we manually created a clean disease lexicon by combining a automatic approach with manual curation. However, there is need to increase the coverage of the underlying disease lexicon [30]. (5) Not all sentences in a document are equally informative. Sentence type is important for assessing the strength of extracted drug-disease associations. For example, the strength of drug-disease treatment is strong if it appears in background section sentences or in conclusion sentences. On the other hand, drug-disease associations in objective sections are weaker. We previously developed an algorithm by combining text classification and hidden Markov modeling techniques to automatically structure MEDLINE abstracts [31]. In the future, we plan to assign a confidence score to each extracted association by taking sentence type into account. (6) Negation detection, or sentimental classification of drug-disease treatment relationships into subtypes is important. Some of the possible subtypes of drug-disease treatment relationships include “effective and safe,” “effective, not safe,” “safe, not effective,” and “not effective.” Examples include “**Metronidazole** proved to be effective and safe in the treatment of **perioral dermatitis** in children.” (PMID 09407169) (“effective and safe”); “Anthracyclines are effective in the treatment of leukemia, but their use is limited because of cardiotoxicity” (PMID 17043024) (“effective, not safe”); “ Etanercept, at the dosage used, was well tolerated but not effective in the treatment of PSC.” (PMID 14992426) (“safe, not effective”); “Azithromycin was not as effective for the treatment of rosacea.”(PMID 15370397) (“not effective”). In addition, for repositioning strategies based on drug-disease treatment similarity, it is necessary to further differentiate palliative treatments from primary treatments. (7) Patient population characteristics (e.g. age, set) are important for better understanding drug-disease treatment relationships. Consider the following sentence “**Forlax** is safe and effective in the treatment of **constipation** in *children over 8 years old*” (PMID 17937851) and “**Lubiprostone** (Amitiza), appears to be effective for the treatment of **chronic constipation** for *elderly patients*” (PMID 18053448).

## Conclusions

We developed a pattern-based biomedical relationship extraction method and extracted 34,305 unique drug-disease pairs from 20 million MEDLINE abstracts. Our algorithm achieved a precision of 0.904 and a recall of 0.131 for all pairs, and a precision of 0.904 and a recall of 0.842 for frequent pairs. We have shown that the extracted drug-disease pairs positively correlate with drug targets as well as therapeutic classes. We demonstrate that the published articles available on MEDLINE are a valuable source of drug-disease treatment information. The pattern-based relationship extraction algorithm is able to accurately extract many additional pairs from MEDLINE. These accurate and machine-understandable drug-disease pairs have high potential in computational drug repurposing tasks.

## Competing interests

The authors declare that they have no competing interests.

## Authors’ contributions

Xu and Wang have jointly conceived the idea, designed and implemented the algorithms, and prepared the manuscript. Both authors read and approved the final manuscript.
